# DIRECTIONALITY BETWEEN COGNITION AND DEPRESSIVE SYMPTOMS: A LONGITUDINAL CROSS-LAGGED PANEL ANALYSIS

**DOI:** 10.1093/geroni/igz038.592

**Published:** 2019-11-08

**Authors:** Randolph Chan, Jennifer Y M Tang, Tianyin Liu, Gloria H Y Wong

**Affiliations:** 1 The Education University of Hong Kong, Hong Kong, Hong Kong; 2 Sau Po Centre on Ageing, The University of Hong Kong, Pok Fu Lam, Hong Kong; 3 The University of Hong Kong, Hong Kong, Hong Kong

## Abstract

Background and Objectives: The relationship between objective and subjective cognitive function and depressive symptoms is complex and potentially multidirectional. This longitudinal prospective study examined the directionality of their relationship among a community sample of older people with no known diagnosis or treatment for dementia or depression. Research Design and Methods: We examined the temporal relationship between objective cognitive functioning, subjective cognitive complaints, and depressive symptoms in 1,814 community-dwelling older people at baseline and one-year follow-up using regression and two-wave cross-lagged panel analyses, after controlling for demographic and health confounders. Results: Cross-lagged analysis showed that depressive symptoms at follow-up were directly predicted by baseline subjective cognitive complaints, but not baseline objective cognitive functioning. The effect differed across objective cognitive functioning levels. In people with clinically significant cognitive impairment at baseline, objective cognitive decline but not baseline subjective cognitive complaints predicted depressive symptoms. In people with mild objective cognitive impairment at baseline, baseline subjective complaints but not objective cognitive decline predicted depressive symptoms. Discussion and Implications: The effects of objective and subjective cognitive decline on depressive symptoms varied across older people with different levels of cognitive impairment. Awareness and insight of one’s cognitive status may contribute to the development/progression in depressive symptom in people with mild cognitive impairment. Mechanisms unrelated to appraisal may be involved in increased depressive symptoms among older persons with significant objective cognitive impairment.

